# A Retrospective Analysis of Nearly 3,000,000 Non-COVID-19 Mid- and High-Priority Ambulance Activations Before and During COVID-19: A National Study from Saudi Arabia

**DOI:** 10.3390/healthcare14183106

**Published:** 2026-09-20

**Authors:** Saeed A. Alqahtani, Talal M. Alshammari, Abdullah M. Alshamrani, Tariq L. Alshabaani, Tarek M. Esmael, Asif A. Mahmood, Abdulmajeed A. Alamri, Ahmed A. Alshamrani, Nawaf H. Alshaye, Abdulatif S. Alamri, Salem R. Aldossary, Yousef M. Alsofayan, Fahad S. Alhajjaj, Jawaher M. Alkhaldi, Ahmad A. Alrawashdeh

**Affiliations:** 1 Department of Emergency Medical Services, Arabian Gulf University, Manama P.O. Box 26671, Bahrain; 2 Department of Emergency Medical Care, Imam Abdulrahman Bin Faisal University, Dammam 31441, Saudi Arabia; 3 Department of Emergency Medical Services, Prince Sultan Military College of Health Sciences, Dhahran 34313, Saudi Arabia; 4 Department of Medical Affairs, Saudi Red Crescent Authority, Riyadh 11129, Saudi Arabia; 5 Directorate of Medical Research, Saudi Red Crescent Authority, Riyadh 11129, Saudi Arabia; 6 Directorate of Military Medical Services, Ministry of Defense, Riyadh 11159, Saudi Arabia; 7 Department of Allied Medical Sciences, Jordan University of Science and Technology, Irbid 22110, Jordan

**Keywords:** COVID-19, EMS, ambulance call, SRCA, Saudi Arabia

## Abstract

**Background/Objectives:** Evidence regarding sustained changes in ambulance utilization during the COVID-19 pandemic is inconsistent, and long-term national evidence from Saudi Arabia is limited. This study assessed changes in the level and weekly trend of eligible non-COVID-19 mid-priority, potentially life-threatening, and life-threatening ambulance activations and EMS time intervals recorded by the Saudi Red Crescent Authority (SRCA) during the COVID-19 period relative to the pre-pandemic period. **Methods:** We retrospectively analyzed eligible SRCA activations recorded between 1 March 2018 and 28 February 2022. Weekly counts were evaluated using segmented negative-binomial interrupted time-series models. Exponentiated coefficients are reported as incidence rate ratios (IRRs) with 95% confidence intervals (CIs). EMS time intervals were compared between periods. **Results:** The analytical cohort comprised 2,837,523 eligible activations. At the interruption on 1 March 2020, the model estimated an immediate 19.0% level increase in weekly call volume (level-change IRR = 1.19; 95% CI: 1.06–1.33), followed by a relative decline in the post-interruption weekly trend (slope-change IRR = 0.996; 95% CI: 0.994–0.997). The largest complaint-specific immediate level-change IRRs were observed for penetrating injuries (IRR = 7.60; 95% CI: 5.40–10.70), pregnancy or obstetric emergencies (IRR = 3.59; 95% CI: 2.66–4.84), and allergic reactions (IRR = 3.21; 95% CI: 2.32–4.44); traffic-accident activations had a lower level (IRR = 0.59; 95% CI: 0.53–0.66). Median response, scene, transport, and total EMS intervals were longer by 1, 2, 2, and 4 min, respectively. **Conclusions:** Among the included non-COVID-19 mid- and high-priority activations, pandemic onset was associated with a higher immediate call level followed by a declining relative weekly trend and modestly longer EMS intervals. Complaint-specific differences may inform surveillance and service-capacity planning, but the observational design and exclusions preclude causal interpretation or inference about total SRCA workload.

## 1. Introduction

In May 2023, the World Health Organization declared that COVID-19 is no longer an international public health emergency, following a death toll of nearly seven million people worldwide [1]. Fear of contracting the disease, curfews, lockdowns, and suspension of elective health services led to a reduction in health-seeking behavior among the public in many regions [2]. While telehealth provided an alternative means of accessing health services during the pandemic, virtual care is designed for non-urgent cases or chronic conditions [3]. For emergency and life-threatening situations, ambulance services serve as the first point of contact for healthcare. The impact of COVID-19 on ambulance services during the pandemic remains unclear. Understanding public utilization of prehospital care services during the pandemic is important for enhancing mitigation, preparedness, response, and recovery strategies. 

Two reviews assessed the impact of COVID-19 on ambulance calls and reported heterogeneous findings across settings and pandemic phases [4,5]. An earlier national SRCA analysis covering January–May 2020 reported increased emergency calls during an early-pandemic comparison period [6]. However, that short descriptive window could not distinguish an immediate interruption from the subsequent longer-term trajectory. Evidence covering extended pre-pandemic and pandemic periods, detailed complaint categories, and operational EMS intervals therefore remains limited.

This study aimed to estimate changes in the immediate level and subsequent weekly trend of eligible non-COVID-19 mid-priority, potentially life-threatening, and life-threatening ambulance activations during the COVID-19 period relative to the pre-pandemic period using national SRCA data. We hypothesized that the level and trajectory of the included activations and EMS time intervals would differ between the two periods.

## 2. Materials and Methods

This national retrospective call-level study covered four years, comprising a two-year pre-pandemic period (1 March 2018–28 February 2020) and a two-year COVID-19 period (1 March 2020–28 February 2022). The study team received a fixed, analysis-ready SRCA extract containing non-COVID-19 ambulance activations classified at dispatch as mid-priority, potentially life-threatening, or life-threatening. Low-priority contacts and suspected or confirmed COVID-19 calls were outside the target cohort, and event-participation missions did not contribute to the analyzed call totals. Low-priority contacts were ordinarily managed through telephone advice, referral, or self-transport rather than immediate ambulance deployment. The upstream source database and exclusion-specific screening counts were not supplied; therefore, counts removed by reason and period and a numerical exclusion flow diagram could not be produced. Accordingly, the study does not estimate total SRCA contacts or total pandemic-related EMS workload. Ethical approval was granted by the SRCA Institutional Review Board (Reference number 22-15E; 5 June 2022). Data were de-identified, and individual consent was waived.

The SRCA is the primary ambulance service provider in Saudi Arabia, serving over 35.3 million people across 13 regions and covering a total area of 2,150,000 km^2^ (www.stats.gov.sa). Each call made through the emergency phone number 997 or smartphone application (Asefni) that requires prehospital care services is recorded, whether or not the caller is transported. For every call classified by the dispatcher as mid-priority, potentially life-threatening, or life-threatening, an ambulance staffed with basic, advanced life support personnel, or both are deployed as appropriate. Low-priority calls are managed through telephone triage by online medical controllers. Callers in this category may be advised to self-transport to a hospital, referred to a general practitioner, or upgraded to a higher priority level as needed. Prehospital care providers operate under the clinical practice guidelines developed by the SRCA.

Between March and June 2020, many government measures were implemented to contain the spread of COVID-19 [7]. These include suspension of Umrah, schools, all social gatherings, sports activities, prayers in mosques, domestic and international flights, public transportation, as well as the imposition of curfews and lockdowns. During this period, Hajj was severely restricted, and many business activities were suspended. By 21 June 2020, the resumption of normal activities began, with continued adherence to social distancing and mandatory facemask wearing. On 10 December 2020, the Pfizer–BioNTech COVID-19 vaccine was approved for use in Saudi Arabia.

Data were obtained from the SRCA electronic database. Dispatchers recorded information received from callers, and attending prehospital clinicians crosschecked and updated the record when a unit attended. Variables included age, sex, call type, call time, transport status, region, primary complaint, and operational timestamps. The analytical unit was an ambulance activation rather than a unique patient; repeated activations involving the same person could not be linked in the de-identified data. Missing values were not statistically imputed. Calls with missing characteristics contributed to the overall series but not to weekly series stratified by that characteristic. Sex was missing in 1,285,015 calls (45.3%): 79.8% before and 18.4% during the COVID-19 period. Age was missing in 1,336,700 calls (47.1%): 81.1% before and 20.7% during the COVID-19 period. Primary complaints were grouped into broader categories; the aggregate penetrating-injury category included stabbing, gunshot injury, and impalement, but mechanism and intent were unavailable.

**Figure 1:**
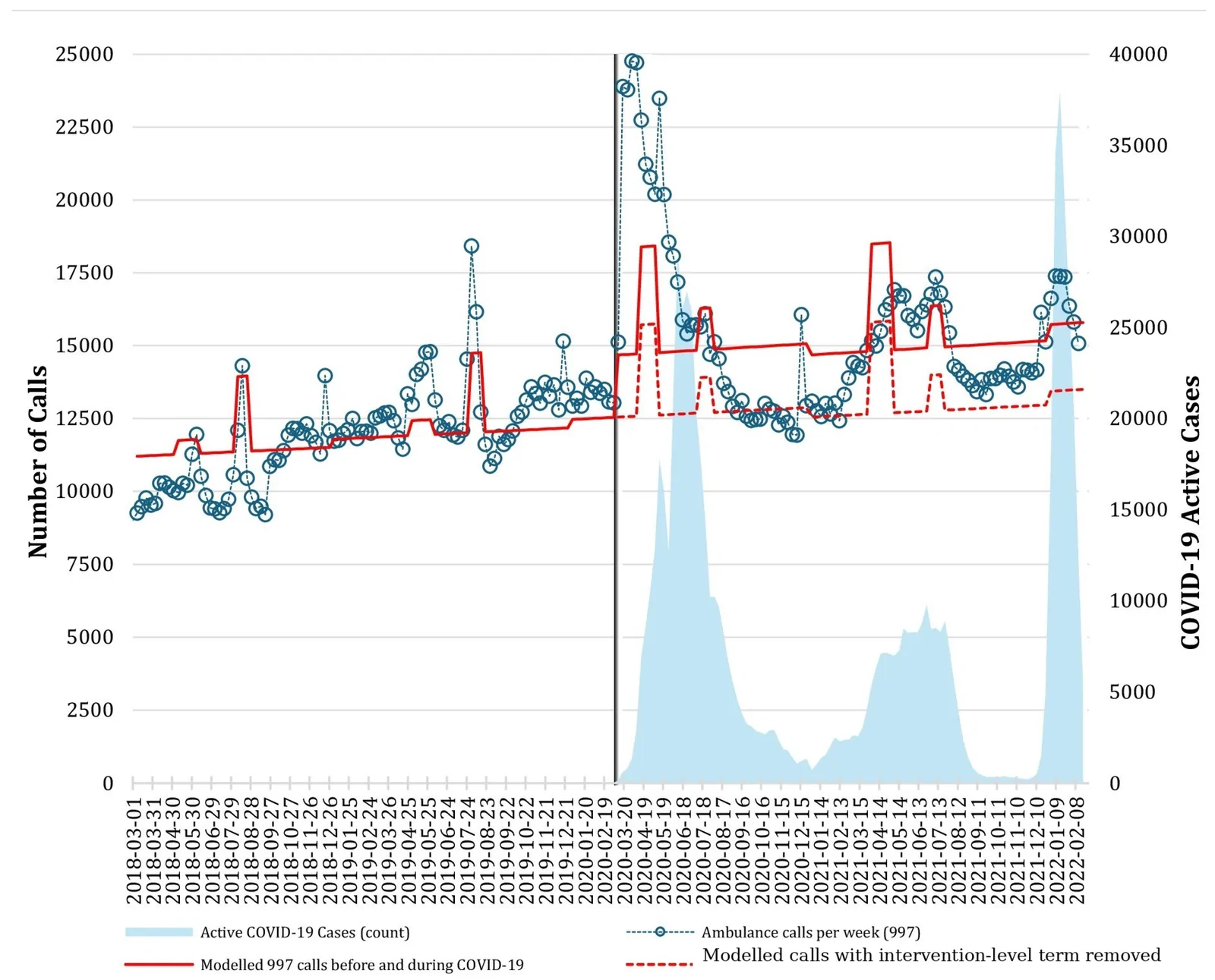
Observed weekly ambulance-call volume, modeled volume before and during COVID-19, active COVID-19 cases (shaded area; right axis), and the dashed fitted series with the intervention-level coefficient removed. The dashed series retains the estimated post-intervention trend, should not be interpreted as a no-pandemic counterfactual, and was not used to estimate excess calls.

Eligible call-level records were aggregated into 207 consecutive weekly counts. Weekly outcomes were analyzed using log-link negative-binomial segmented regression. Predictors included continuous study time in weeks, a binary pandemic-interruption indicator, and a post-interruption time term representing the change in weekly trend. The exponentiated interruption-indicator coefficient was interpreted as the immediate level change at pandemic onset, whereas the exponentiated post-interruption time coefficient represented the relative change in weekly slope. Hajj was represented by three consecutive weekly indicators per study year and Ramadan by five, with separate coefficients estimated before and during the pandemic period. The logarithm of the estimated annual Saudi residential population was included as an offset, and Newey–West standard errors were used to account for autocorrelation. The overall series and exploratory series stratified by sex, age, call type, call time, transport status, region, and 25 primary-complaint categories were fitted independently. The national population offset in the stratified models means these coefficients describe national-population-adjusted call volume within recorded categories, not incidence within a sex, age group, or region, and they are not weighted components of the overall IRR. Estimates are reported as IRRs with 95% CIs. In Figure 1, the dashed fitted series was generated by removing the intervention-level coefficient while retaining the estimated post-intervention trend and the other model terms; it is not a no-pandemic counterfactual and was not used to estimate excess calls.

EMS intervals were derived from SRCA-recorded operational timestamps. Response time was the recorded call-receipt-to-scene-arrival interval when available; otherwise, the supplied cleaning rule used dispatch-to-scene time plus three minutes. Scene time was scene arrival to scene departure, transport time was scene departure to receiving-hospital arrival, and total EMS time was calculated at the record level from the component intervals. Available recorded values were analyzed without statistical imputation or an additional interval-magnitude exclusion. Medians and interquartile ranges (IQRs) were compared using the Wilcoxon rank-sum test. Two-tailed *p* < 0.05 was considered statistically significant. Analyses used Stata version 16.0 (StataCorp, College Station, TX, USA). The fixed response-time reconstruction was applied to 695,664 of 2,837,424 records contributing to the response-time analysis (24.5%): 347,977 of 1,241,799 (28.0%) before and 347,687 of 1,595,625 (21.8%) during the COVID-19 period. Of these replacements, 694,636 followed a missing directly recorded call-receipt-to-scene interval and 1028 followed a negative interval. This was a deterministic timestamp-reconstruction rule rather than model-based statistical imputation.

## 3. Results

The final analytical cohort comprised 2,837,523 eligible ambulance activations between 1 March 2018 and 28 February 2022; 2,825,947 calls contributed to the complete-week ITS series, including 1,257,314 before and 1,568,633 during the COVID-19 period. Average weekly volume was approximately 12,000 before the interruption and 15,400 during the COVID-19 period. On 1 March 2020, the model estimated an immediate 19.0% level increase (level-change IRR = 1.19; 95% CI: 1.06–1.33). This was followed by a relative decline in the post-interruption weekly trend compared with the pre-pandemic trend (slope-change IRR = 0.996; 95% CI: 0.994–0.997). The level-change IRR should not be interpreted as an average 19.0% increase throughout the complete two-year pandemic period. Observed and fitted weekly call volumes are shown in Figure 1. 

### 3.1. Impact of COVID-19 on Ambulance Calls Across Key Response Elements 

Table 1 presents exploratory estimates across recorded call characteristics. Sex and age-group estimates are retained for transparency but are not interpreted because demographic completeness differed markedly between periods. Among the other recorded characteristics, higher immediate level-change estimates were observed for trauma calls, nighttime calls, non-transported cases, and 11 regions. No statistically significant immediate level change was observed for medical calls, daytime calls, transported cases, Al-Madinah, or Makkah. Because these series were fitted independently with the national population offset, they should not be interpreted as a decomposition of the overall estimate or as within-subgroup incidence.

### 3.2. Impact of COVID-19 on Ambulance Calls Stratified by the Primary Complaint

**Figure 2:**
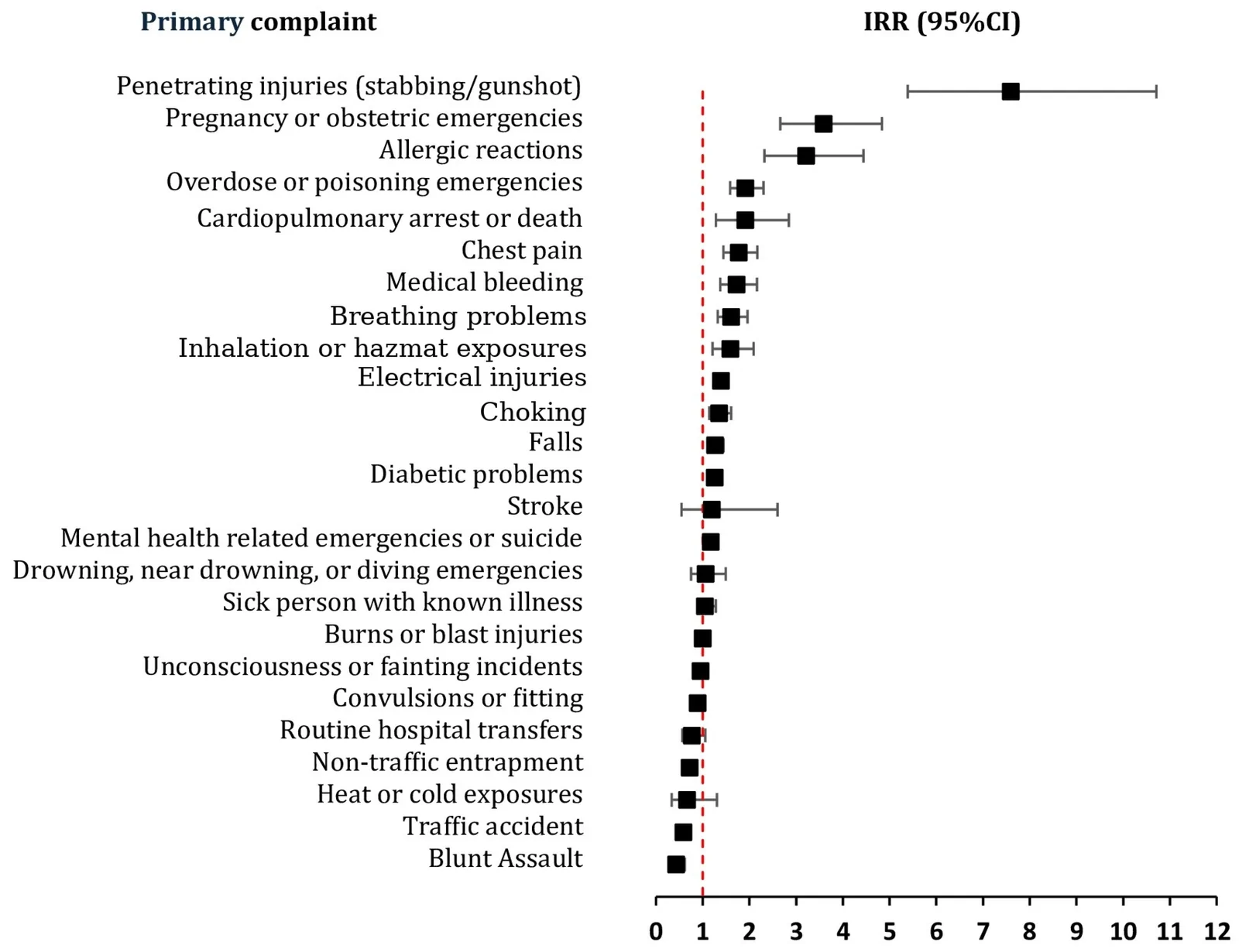
Estimated immediate level change in weekly eligible ambulance activations on 1 March 2020, stratified by primary complaint. Squares show IRRs and horizontal lines show 95% CIs.

Figure 2 presents exploratory immediate level-change estimates for the 25 primary-complaint categories. Fourteen categories had higher level-change estimates, four had lower estimates, and seven showed no statistically significant level change. The largest relative estimates were observed for penetrating injuries, pregnancy or obstetric emergencies, and allergic reactions; the lowest were observed for blunt assault and traffic accidents. In the complete-week series, the unadjusted pre-/during-period counts were 40,645/195,797 for penetrating injuries, 9992/20,393 for pregnancy or obstetric emergencies, 2542/4387 for allergic reactions, and 232,796/205,354 for traffic accidents. These descriptive counts are not substitutes for the adjusted ITS estimates and do not establish the absolute contribution of each category to the overall change.

Figure 2 provides the visual comparison of immediate level changes, whereas Table 2 provides the corresponding numerical level- and post-interruption slope-change estimates. Post-interruption weekly trends declined for most complaint categories; increases were estimated for heat or cold exposure, blunt assault, drowning or diving emergencies, traffic accidents, and convulsions or fitting.

### 3.3. EMS Time Intervals Before and During the COVID-19 Period

Median response, scene, transport, and total EMS intervals were longer during the COVID-19 period by 1, 2, 2, and 4 min, respectively (all *p* < 0.001; Table 3). Given the large analytical sample, these *p*-values should be interpreted alongside the modest absolute differences; clinical consequences cannot be inferred because patient outcomes were unavailable.

## 4. Discussion

This national interrupted time-series analysis found an immediate 19.0% level increase in the included non-COVID-19 mid- and high-priority ambulance activations on 1 March 2020, followed by a relative decline in the post-interruption weekly trend. These coefficients are interpreted separately: the level-change IRR represents the estimated immediate increase at onset and not an average 19.0% increase throughout the two-year pandemic period. Penetrating injuries, pregnancy or obstetric emergencies, and allergic reactions had the largest relative level-change estimates, but the available data do not establish that these categories caused or drove the overall change. Median record-level total EMS time was four minutes longer during the COVID-19 period.

The effect of the pandemic on EMS demand varied across settings. In US national data, total 911 EMS activations decreased after March 2020, although the proportions involving on-scene death, cardiac arrest, and opioid use or overdose increased [8]. In a UK national survey, weekly call volume temporarily peaked at 13.1% above baseline in week 7, but all participating services ended the study period below baseline [9]. An earlier national SRCA study reported increased emergency calls during an early-pandemic comparison [6]. The present study extends that evidence by using four years of weekly data and distinguishing the estimated immediate level change from the subsequent slope change. Findings from Berlin, Victoria, and Vaud also varied [10,11,12], reinforcing the importance of study period, case definition, and EMS context. Similar heterogeneity has been described in other regional EMS studies [13,14,15,16,17].

Sex- and age-stratified estimates are retained in Table 1 for transparency but are not interpreted because demographic recording improved substantially over time. More generally, the exploratory stratified models were fitted independently and used the national population offset; they therefore describe changes in category-specific national call burden rather than within-group incidence and cannot be combined to explain the overall coefficient. Changes in case mix, missingness, coding, mobility, access to other services, and non-transport patterns may all have contributed.

Saudi hospital-based and international studies reported changes in trauma and assault patterns during pandemic restrictions, but they did not demonstrate a nationwide change directly comparable with the SRCA aggregate penetrating-injury category [18,19]. Pandemic-related psychosocial stress has been proposed as a general pathway for interpersonal aggression [20], but the present data cannot test that mechanism. In the complete-week series, penetrating-injury calls numbered 40,645 before and 195,797 during the pandemic period, with an immediate level-change IRR of 7.60 (95% CI: 5.40–10.70). The category combined stabbing, gunshot injury, and impalement; mechanism and intent were unavailable. Misclassification or contemporaneous coding changes cannot be excluded, and firearm-specific attribution is not supported. Pregnancy or obstetric-emergency calls numbered 9992 and 20,393, respectively, with an immediate level-change IRR of 3.59 (95% CI: 2.66–4.84). Prior hospital-based studies reported changes in obstetric and gynecological emergency use during the pandemic [21], but the present data do not identify the clinical condition, reason for ambulance use, or final outcome.

Allergic-reaction calls numbered 2542 before and 4387 during the pandemic period, with an immediate level-change IRR of 3.21 (95% CI: 2.32–4.44). Although allergic disease, vaccine adverse events, and anaphylaxis trends have been described in other populations [22,23,24,25,26], these outcomes are not equivalent to ambulance calls coded as allergic reactions. Vaccination status, suspected allergen, severity, temporal relationship, and final diagnosis were unavailable. The present analysis therefore cannot attribute the change to COVID-19 vaccination or any other specific exposure.

Longer EMS intervals during the pandemic have also been reported in South Korea, Iran, and Poland [27,28,29]. In this study, the median response-time difference was one minute, scene and transport times were each two minutes longer, and record-level total time was four minutes longer. These statistically precise but modest operational differences should not be interpreted as evidence of clinical harm because patient outcomes and validated clinically important thresholds were unavailable. Higher call volume and reduced unit availability may have created dispatch queues, as described by bottleneck theory [4]. Infection-control procedures, scene-management requirements, and receiving-hospital constraints may also have contributed. These mechanisms were not directly measured and remain plausible explanations rather than demonstrated causes.

The findings support flexible ambulance-capacity planning and continued surveillance of complaint-specific and operational patterns during prolonged emergencies. The large relative change in the aggregate penetrating-injury category warrants mechanism-specific verification rather than attribution to firearm availability or a particular event. Likewise, pregnancy or obstetric-emergency findings warrant linkage with hospital diagnoses and outcomes. Although women face documented barriers to healthcare access across income settings [30,31], these call-level data cannot establish whether pandemic restrictions caused the observed change. Future studies should link dispatch, clinical, transport, and hospital-outcome data. National resource planning should also consider the operational context described in ambulance-service and health-transformation reports [32,33,34].

This study has several limitations. First, the retrospective observational design and absence of a concurrent control series preclude causal attribution to COVID-19 or any particular policy. The single interruption on 1 March 2020 summarized a heterogeneous two-year period and did not separately model lockdown, reopening, successive infection waves, service changes, or vaccination. Other time-varying factors may have affected the estimates. Second, the analysis was restricted to the SRCA-provided eligible extract and does not represent total EMS workload; upstream exclusion-specific counts were unavailable. Third, repeated activations involving the same person could not be identified. Sex was missing in 45.3% and age in 47.1% of calls overall, with substantially greater missingness before than during the pandemic period. The retained demographic estimates may therefore reflect changes in documentation as well as call volume and should be considered exploratory. Routine complaint categories may also be misclassified. Fourth, the national population offset estimates category-specific national call burden rather than within-subgroup incidence, and independently fitted subgroup estimates are not components of the overall IRR. Fifth, multiple exploratory models increase the possibility of chance findings. Sixth, hospital admission, final diagnosis, mortality, and other clinical outcomes were unavailable, so clinical consequences could not be assessed. Penetrating-injury mechanism and intent and the causes of allergic reactions were unavailable. Finally, rare implausible or extreme timestamps remained in the routine data; medians reduce their influence, but interval data quality could not be independently validated. The findings reflect the Saudi EMS structure and may not generalize to systems with different populations, dispatch arrangements, resources, or healthcare access [35]. Because the reconstruction rule was used more frequently before than during COVID-19 (28.0% vs. 21.8%), the observed one-minute median response-time difference could have been influenced by differential use of this rule and should be interpreted cautiously.

## 5. Conclusions

At the interruption on 1 March 2020, eligible non-COVID-19 mid- and high-priority SRCA activations showed an immediate 19.0% level increase followed by a relative decline in the post-interruption weekly trend. Complaint-specific analyses identified substantial relative differences, but their causes could not be established. Median record-level total EMS time was four minutes longer during the COVID-19 period. These findings support continued national EMS surveillance and flexible capacity planning, while the observational design, exclusions, demographic missingness, and unavailable mechanism and outcome data preclude causal interpretation or inference about total SRCA demand.

## Figures and Tables

**Table 1:** Weekly impact of COVID-19 on ambulance calls.

Response Elements	Immediate Level-Change IRR (95% CI)	Weekly Slope-Change IRR (95% CI)
**Gender**		
Male	1.85 (1.67, 2.04)	0.989 (0.987, 0.991)
Female	2.82 (2.51, 3.18)	0.995 (0.993, 0.997)
**Age groups**		
0–15	2.62 (2.30, 2.98)	0.986 (0.984, 0.988)
16–30	2.05 (1.78, 2.36)	0.989 (0.986, 0.991)
31–45	2.54 (2.24, 2.88)	0.987 (0.985, 0.990)
46–60	2.65 (2.22, 3.17)	0.993 (0.989, 0.996)
61–75	2.25 (1.85, 2.73)	0.995 (0.992, 0.999)
>75	1.88 (1.57, 2.25)	0.992 (0.989, 0.995)
**Type of call**		
Medical	1.09 (0.93, 1.27)	0.996 (0.993, 0.998)
Trauma	1.42 (1.35, 1.50)	0.996 (0.995, 0.997)
**Time of call**		
Daytime (5 AM–6 PM)	1.05 (0.96, 1.15)	0.997 (0.995, 0.998)
Nighttime (6 PM–5 AM)	1.33 (1.16, 1.51)	0.995 (0.993, 0.997)
**Transport status**		
Transported	0.95 (0.83, 1.09)	0.997 (0.995, 0.999)
Not transported	1.55 (1.43, 1.68)	0.994 (0.993, 0.995)
**Region**		
Al-Jouf	1.69 (1.38, 2.10)	0.995 (0.992, 0.998)
Al-Baha	1.59 (1.33, 1.91)	0.994 (0.991, 0.996)
Jizan	1.57 (1.35, 1.83)	0.991 (0.989, 0.994)
Najran	1.57 (1.24, 1.99)	0.994 (0.990, 0.997)
Asir	1.47 (1.31, 1.66)	0.995 (0.993, 0.997)
Dammam	1.40 (1.22, 1.61)	0.994 (0.992, 0.996)
Arar	1.39 (1.18, 1.63)	0.994 (0.992, 0.996)
Hail	1.36 (1.25, 1.49)	0.998 (0.997, 0.999)
Al-Qassim	1.33 (1.19, 1.49)	0.997 (0.995, 0.999)
Al-Riyadh	1.23 (1.12, 1.35)	1.000 (0.999, 1.001)
Tabuk	1.16 (1.02, 1.32)	0.999 (0.997, 1.001)
Al-Madinah	1.10 (0.96, 1.26)	0.998 (0.996, 1.001)
Makkah	0.92 (0.78, 1.10)	0.991 (0.988, 0.995)

Abbreviations: IRR, incidence rate ratio; CI, confidence interval. Sex- and age-stratified estimates are exploratory and include only calls with recorded demographic information. Sex was missing in 45.3% of calls (79.8% before and 18.4% during the COVID-19 period), and age was missing in 47.1% (81.1% and 20.7%, respectively). Because completeness differed markedly between periods, these estimates may reflect changes in documentation as well as call volume and should not be interpreted as subgroup-specific incidence or as weighted components of the overall IRR.

**Table 2:** Immediate level-change and post-interruption weekly slope-change IRRs for eligible ambulance activations, by primary complaint.

No	Primary Complaint	Immediate Level-Change IRR (95% CI)	Weekly Slope-Change IRR (95% CI)
1	Penetrating injuries	7.60 (5.40, 10.70)	0.983 (0.976, 0.989)
2	Pregnancy or obstetric emergencies	3.59 (2.66, 4.84)	0.993 (0.988, 0.997)
3	Allergic reactions	3.21 (2.32, 4.44)	0.992 (0.987, 0.997)
4	Overdose or poisoning emergencies	1.91 (1.58, 2.30)	0.993 (0.990, 0.996)
5	Cardiopulmonary arrest or death	1.90 (1.28, 2.84)	0.973 (0.965, 0.981)
6	Chest pain	1.76 (1.44, 2.17)	0.994 (0.991, 0.997)
7	Medical bleeding	1.72 (1.37, 2.16)	0.990 (0.987, 0.993)
8	Breathing problems	1.60 (1.32, 1.95)	0.994 (0.991, 0.997)
9	Inhalation or hazmat exposures	1.59 (1.21, 2.09)	0.998 (0.993, 1.002)
10	Electrical injuries	1.38 (1.26, 1.52)	1.000 (0.999, 1.002)
11	Choking	1.35 (1.13, 1.60)	0.993 (0.990, 0.996)
12	Falls	1.26 (1.11, 1.44)	0.996 (0.994, 0.998)
13	Diabetic problems	1.26 (1.15, 1.37)	0.996 (0.995, 0.998)
14	Stroke	1.19 (0.54, 2.60)	0.952 (0.934, 0.970)
15	Mental health emergencies or suicide	1.16 (1.09, 1.24)	0.993 (0.992, 0.994)
16	Drowning or diving emergencies	1.05 (0.75, 1.49)	1.007 (1.001, 1.013)
17	Sick person with known illness	1.04 (0.90, 1.20)	0.992 (0.990, 0.995)
18	Burns or blast injuries	1.00 (0.92, 1.10)	0.993 (0.991, 0.994)
19	Unconsciousness or fainting incidents	0.95 (0.89, 1.01)	0.999 (0.998, 1.000)
20	Convulsions or fitting	0.89 (0.84, 0.94)	1.001 (1.000, 1.001)
21	Routine hospital transfers	0.76 (0.56, 1.05)	0.995 (0.990, 1.001)
22	Non-traffic entrapment	0.71 (0.69, 0.74)	1.000 (0.999, 1.001)
23	Heat or cold exposures	0.66 (0.33, 1.30)	1.029 (1.018, 1.039)
24	Traffic accidents	0.59 (0.53, 0.66)	1.004 (1.002, 1.006)
25	Blunt assault	0.43 (0.31, 0.61)	1.022 (1.016, 1.029)

Abbreviations: IRR, incidence rate ratio; CI, confidence interval. Complaint-specific series were fitted independently using the national population offset; estimates describe category-specific national call burden and should not be interpreted as within-category incidence or as components of the overall IRR.

**Table 3:** EMS time intervals in minutes before and during the COVID-19 period.

	Overall	Before-COVID-19	During COVID-19	*p*-Value
EMS time intervals, minutes, median (IQR) ^a^				
Response time	14 (10, 20)	14 (10, 20)	15 (11, 21)	<0.001
Scene time	17 (11, 26)	16 (9, 25)	18 (12, 27)	<0.001
Transport time	13 (8, 21)	12 (7, 21)	14 (8, 22)	<0.001
Total EMS time	47 (34, 63)	45 (32, 61)	49 (36, 64)	<0.001

^a^ Available n: response, 2,837,424; scene, 2,837,370; transport, 1,579,752; and total, 1,579,750. Transport and total intervals were available mainly for transported calls. The deterministic response-time reconstruction described in Section 2 was used for 695,664/2,837,424 response-time records (24.5%).

## Data Availability

The data sets used and analyzed during the current study are available from the corresponding authors on reasonable requests.

## Abbreviations

The following abbreviations are used in this manuscript:

EMS	Emergency Medical Services
IRR	Incidence Rate Ratio
SRCA	Saudi Red Crescent Authority
